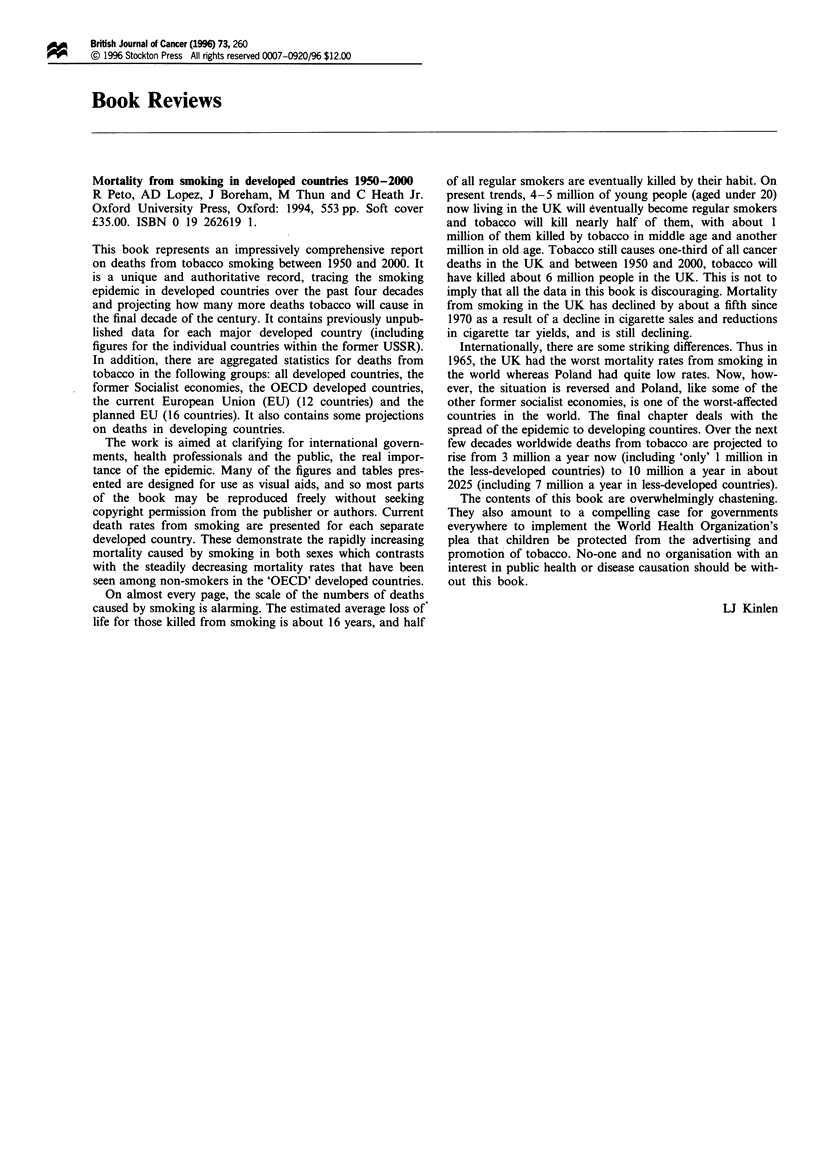# Mortality from smoking in developed countries 1950-2000

**Published:** 1996-01

**Authors:** LJ Kinlen


British Journal of Cancer (1996) 73, 260
© 1996 Stockton Press All rights reserved 0007-0920/96 $12.00

### Book Reviews

**Mortality from smoking in developed countries 1950–2000**
R Peto, AD Lopez, J Boreham, M Thun and C Heath Jr.
Oxford University Press, Oxford: 1994, 553 pp. Soft cover £35.00. ISBN 0 19 262619 1.

This book represents an impressively comprehensive report on deaths from tobacco smoking between 1950 and 2000. It is a unique and authoritative record, tracing the smoking epidemic in developed countries over the past four decades and projecting how many more deaths tobacco will cause in the final decade of the century. It contains previously unpublished data for each major developed country (including figures for the individual countries within the former USSR). In addition, there are aggregated statistics for deaths from tobacco in the following groups: all developed countries, the former Socialist economies, the OECD developed countries, the current European Union (EU) (12 countries) and the planned EU (16 countries). It also contains some projections on deaths in developing countries.

The work is aimed at clarifying for international governments, health professionals and the public, the real importance of the epidemic. Many of the figures and tables presented are designed for use as visual aids, and so most parts of the book may be reproduced freely without seeking copyright permission from the publisher or authors. Current death rates from smoking are presented for each separate developed country. These demonstrate the rapidly increasing mortality caused by smoking in both sexes which contrasts with the steadily decreasing mortality rates that have been seen among non-smokers in the 'OECD' developed countries.

On almost every page, the scale of the numbers of deaths caused by smoking is alarming. The estimated average loss of life for those killed from smoking is about 16 years, and half of all regular smokers are eventually killed by their habit. On present trends, 4–5 million of young people (aged under 20) now living in the UK will eventually become regular smokers and tobacco will kill nearly half of them, with about 1 million of them killed by tobacco in middle age and another million in old age. Tobacco still causes one-third of all cancer deaths in the UK and between 1950 and 2000, tobacco will have killed about 6 million people in the UK. This is not to imply that all the data in this book is discouraging. Mortality from smoking in the UK has declined by about a fifth since 1970 as a result of a decline in cigarette sales and reductions in cigarette tar yields, and is still declining.

Internationally, there are some striking differences. Thus in 1965, the UK had the worst mortality rates from smoking in the world whereas Poland had quite low rates. Now, however, the situation is reversed and Poland, like some of the other former socialist economies, is one of the worst-affected countries in the world. The final chapter deals with the spread of the epidemic to developing countires. Over the next few decades worldwide deaths from tobacco are projected to rise from 3 million a year now (including 'only' 1 million in the less-developed countries) to 10 million a year in about 2025 (including 7 million a year in less-developed countries).

The contents of this book are overwhelmingly chastening. They also amount to a compelling case for governments everywhere to implement the World Health Organization's plea that children be protected from the advertising and promotion of tobacco. No-one and no organisation with an interest in public health or disease causation should be without this book.

LJ Kinlen